# Enrichment of Immune Dysregulation Disorders in Adult Patients with Human Inborn Errors of Immunity

**DOI:** 10.1007/s10875-024-01664-2

**Published:** 2024-02-16

**Authors:** Alejandro Segura-Tudela, Marta López-Nevado, Celia Nieto-López, Sandra García-Jiménez, María J. Díaz-Madroñero, Ángeles Delgado, Oscar Cabrera-Marante, Daniel Pleguezuelo, Pablo Morales, Estela Paz-Artal, Jorge Gil-Niño, Francisco M. Marco, Cristina Serrano, Luis I. González-Granado, Juan F. Quesada-Espinosa, Luis M. Allende

**Affiliations:** 1 Department of Immunology, University Hospital, 12 de Octubre, Avda. de Andalucía S/N, 28041 Madrid, Spain; 2 Research Institute Hospital, 12 Octubre (imas12), Madrid, Spain; 3 School of Medicine, Complutense University of Madrid, Madrid, Spain; 4 Centro de Investigación Biomédica en Red de Enfermedades Infecciosas, Instituto de Salud Carlos III, Madrid, Spain; 5 Department of Internal Medicine, University Hospital, 12 de Octubre, Madrid, Spain; 6 Unit of Immunology, University Hospital General Dr Balmis, Alicante, Spain; 7 Department of Immunology, University Hospital Fundación Jiménez Díaz, Madrid, Spain; 8 Unit of Immunodeficiencies, Department of Pediatrics, University Hospital, 12 de Octubre, Madrid, Spain; 9 Department of Genetics, University Hospital, 12 de Octubre, Madrid, Spain

**Keywords:** Autoimmune lymphoproliferative syndrome (ALPS), autoimmunity, germline variants, human inborn errors of immunity (IEI), infections, Jeffrey model foundation warning signs, immune dysregulation, lymphoproliferation, NGS, primary immune regulatory disorders (PIRD), somatic variants

## Abstract

Human inborn errors of immunity (IEI) comprise a group of diseases resulting from molecular variants that compromise innate and adaptive immunity. Clinical features of IEI patients are dominated by susceptibility to a spectrum of infectious diseases, as well as autoimmune, autoinflammatory, allergic, and malignant phenotypes that usually appear in childhood, which is when the diagnosis is typically made. However, some IEI patients are identified in adulthood due to symptomatic delay of the disease or other reasons that prevent the request for a molecular study. The application of next-generation sequencing (NGS) as a diagnostic technique has given rise to an ever-increasing identification of IEI-monogenic causes, thus improving the diagnostic yield and facilitating the possibility of personalized treatment. This work was a retrospective study of 173 adults with IEI suspicion that were sequenced between 2005 and 2023. Sanger, targeted gene-panel, and whole exome sequencing were used for molecular diagnosis. Disease-causing variants were identified in 44 of 173 (25.43%) patients. The clinical phenotype of these 44 patients was mostly related to infection susceptibility (63.64%). An enrichment of immune dysregulation diseases was found when cohorts with molecular diagnosis were compared to those without. Immune dysregulation disorders, group 4 from the International Union of Immunological Societies Expert Committee (IUIS), were the most prevalent among these adult patients. Immune dysregulation as a new item in the Jeffrey Model Foundation warning signs for adults significantly increases the sensitivity for the identification of patients with an IEI-producing molecular defect.

**Supplementary Information:**

The online version contains supplementary material available at 10.1007/s10875-024-01664-2.

## Introduction

Inborn errors of immunity (IEI), previously known as primary immunodeficiencies, are a large group of heterogeneous diseases originating from molecular alterations in genes related to the innate or adaptive immune system. Patients with these alterations develop an increased susceptibility to infectious diseases (mediated by viruses, bacteria, or fungi) and/or a growing diversity of autoimmune, autoinflammatory, allergic, bone marrow failure, or malignant phenotypes. Although IEI are considered rare diseases, the number of this type of disorders has been growing year by year, and more than 485 genetic defects related to IEI are currently described. The International Union of Immunological Societies (IUIS) categorizes IEI into nine main groups and an extra 10th group for IEI-phenocopies. These groups are based on overlapping phenotypes shown by different patients [1, 2].

Due to the genetic origin of most of these diseases, symptoms usually appear at the first stages of life and diagnosis is commonly made during childhood. This early diagnosis allows the establishment of a tailored treatment and appropriate genetic counseling to achieve considerable improvement and even cure these patients. Nevertheless, some IEI patients with pathologic variants are diagnosed in adulthood because of a late onset of symptoms [3] or nonspecific multisystemic presentations that finally give rise to a delay in ordering specific molecular studies for the diagnosis of IEI. There are different hypotheses to explain the symptomatic delay of these diseases, such as contact with a specific environmental trigger, the low penetrance of some hypomorphic variants, the development of epigenetic modifications of specific genes, or the presence of somatic variants [3].

An immunophenotype-based approach followed by Sanger sequencing was the most common strategy to reach a genetic diagnosis of IEI until approximately a decade ago. However, there were difficulties in finding an accurate diagnosis of patients using this strategy, such as the large number of IEI-related genes, high variability in the genotype–phenotype relationship, or similar phenotypes shared by different genotypes. Furthermore, Sanger sequencing of multiple candidate genes is a time-consuming, inefficient, and expensive methodology [4]. The growing incorporation of next-generation sequencing (NGS) in hospital environments has allowed a better recognition of previously undiagnosed IEI, not only in infants but also in adult patients [3, 4]. NGS is a sensitive and cost-effective sequencing technology that can be used as a first-line molecular study to identify IEI [5]. The improvement in diagnostic efficiency is clearly demonstrated in some patients with atypical presentations of known IEI [6]. In spite of the benefits achieved by NGS, the identification of disease-causing genetic defects in IEI patients is still a challenge and the diagnostic yield barely reaches 50% in the best of circumstances [5, 7, 8]. Additionally, IEI could fit with other more complex genetic scenarios such as polygenic, epigenetic, or even somatic; not only monogenic models should be considered [9].

In this work, we show the compiled experience in molecular IEI-diagnosis of adult patients between 2005 and 2023. We report on our performance ability to identify IEI in adults, the different types of IEI identified, and the most frequent ages of onset of these pathologies. In addition, we show the most frequent clinical features associated with the detection of IEI in adult patients. We also relate the advantages of a modified list of warning signs for adult patients based on the Jeffrey Model Foundation 10 warning signs to anticipate the presence of an IEI- associated molecular defect.

## Materials and Methods

This work is a retrospective study conducted in the University Hospital 12 de Octubre between 2005 and 2023. Inclusion criteria were adult patients older than 18 years old with suspected IEI that presented clinically as increased susceptibility to infections, autoimmunity, autoinflammatory diseases, allergy, bone marrow failure, and/or malignancy for which their physicians required a molecular study. All patients provided written informed consent in accordance with the Declaration of Helsinki and as approved by the Institutional Research Ethics Committees of University Hospital 12 de Octubre.

Primary clinical manifestations and genetic analysis results were collected and analyzed. Molecular diagnosis was made through Sanger sequencing from 2005 to 2015 (11 years). PCR was used to amplify the coding sequence and the flanking regions of the candidate genes using specific primers. Double-strand DNA templates were sequenced using the dideoxy chain-terminator method of Sanger using an ABI PRISM 3130 genetic analyzer (Thermo Fisher Scientific, Waltham, MA, USA). NGS was performed from 2016 to 2023 (7 ½ years), and two sequencing approaches were used. In the first approach, an in-house-designed targeted 192-gene panel (from January, 2016, to December, 2021) or 434-gene panel (from January, 2022, until June, 2023) (Tables S1 and S2) involved in IEI was sequenced in an Ion Torrent PGM (Thermo Fisher Scientific, Waltham, MA, USA) or MiSeq platform (Illumina, San Diego, CA, USA), respectively. In the second approach, whole exome sequencing (WES) was carried out in cases with atypical clinical and laboratory phenotypes or a negative result in a previous molecular study by Sanger sequencing or the 192/434 gene panels (Table S3). The sequencing workflow followed by our center is shown in Fig. S1. An xGen Exome Panel v1.0 kit (Integrated DNA Technologies, Coralville, IO, USA) and paired-end sequencing (2 × 75 bp) was conducted on a NextSeq 550 (Illumina, San Diego, CA, USA). Bioinformatic analysis was performed using the Karma tool, an in-house pipeline integrating BWA (v0.7.17) and Bowtie2 (v2.4.1) for sequence alignment to the reference genome (hg19 assembly), GATK (v4.1), and VarDict (v1.7.0) for genotyping, ExomeDepth (v.1.10) for CNV detection, AnnotSv (v2.4) for CNV annotation, and ANNOVAR (v2018Apr16) for variant annotation. Karma follows the validation recommendations of the Association for Molecular Pathology (AMP) [10]. Genetic variants were filtered according to alignment and genotyping quality metrics. Variants identified from alignments with low mapping quality, variants with strand bias, variants with a frequency greater than 3% in the gnomAD population database (v2.1.1) [11], and variants classified as benign and probably benign according to the ClinVar database were not evaluated. Clinical interpretation and final classification of the identified variants were performed using information extracted from the gnomAD population database; the genetic variant databases ClinVar, Leiden Open Variant Database (LOVD), and Human Gene Mutation Database (HGMD); the protein domain and structure databases Uniprot, PFAM, and Prosite; and the hereditary disease databases OMIM, Orphanet, and GeneReviews. Variants were classified as pathogenic, likely pathogenic, and variants of uncertain significance (VUS) according to the recommendations of the American College of Medical Genetics and Genomic (ACMG) [12]. Variant filtering and prioritization were performed based on a 455-gene custom panel targeted (Table S3) at the clinical suspicion of IEI. The human genomics community VarSome [13] was used for a comprehensive interpretation of the variants. Candidate gene variants were confirmed by PCR and Sanger sequencing using the same strategy described previously.

Furthermore, copy number variants (CNVs), a 60K chromosomal microarray (CMA) (60K KaryoNIM®, NIMGenetics, Madrid, Spain), were carried out in cases where a heterozygous variant was detected and the form of the disease was autosomal recessive (AR). Analysis and interpretation of the results were performed using Cytogenomics (v.4.0.3.12, Agilent) software. A threshold of ≥ 5 consecutive probes was established to consider a CNV. CNVs detected were classified following previous CMA recommendations for clinical practice. For fluorescence in situ hybridization (FISH), metaphase chromosome spreads were prepared from peripheral blood lymphocytes harvested routinely, and FISH was performed using a commercially available probe, LSI Tuple 1 (Vysis, Abbott), according to the manufacturer’s protocols.

Statistical tests were performed using GraphPad Prism version 7 (GraphPad Prism Software, La Jolla, CA, USA) and RStudio (Posit team (2022); RStudio: Integrated Development Environment for R. Posit Software, PBC, Boston, MA. URL http://www.posit.co/). Statistical significance was calculated by the chi-square test. Sensitivity and specificity were compared by the exact binomial test. Predictive values were compared using the weighted generalized score statistic proposed by Kosinski [14]. Significance was considered only when *P* values were less than 0.05 (*p < 0.05; **p < 0.001).

## Results

### Patient Characteristics and Enrichment of Immune Dysregulation Diseases

A molecular study was requested for 173 adult patients with clinical and laboratory suspicion of IEI. The age of the patients (*n* = 173) ranged from 19 to 85 years, with a mean of 45.94 years. Of the 173 patients who underwent the genetic study, 94 were female (54.34%) and 79 were male (45.66%), which is a sex ratio of 1.19 (Table S4).

**Fig. 1:**
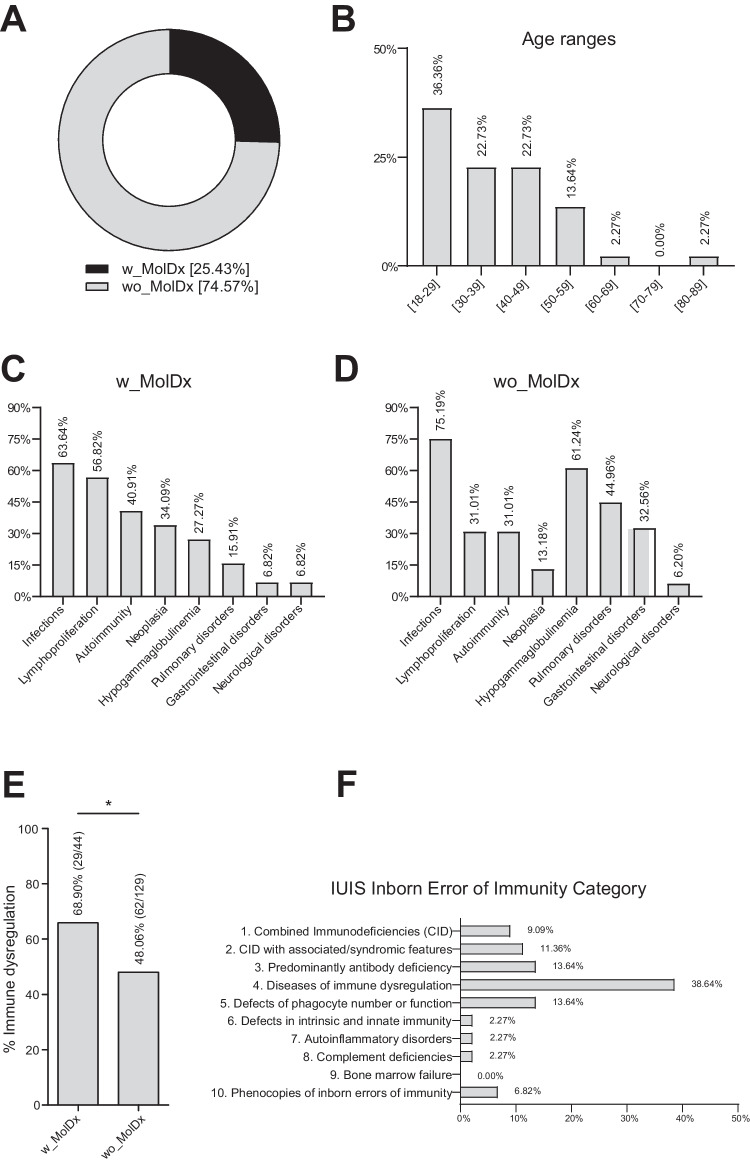
**A** Forty-four patients (25.43%) were molecularly diagnosed (w_MolDx) and 129 patients (74.57%) were not diagnosed (wo_MolDx) in the IEI adult cohort (*n* = 173 patients).** B** Positive genetic diagnosis as a function of age**. C** Clinical features of adult patient w_MolDx. **D** Clinical features of adult patient wo_MolDx. **E** Percentage and number of patients with immune dysregulation according to molecular diagnosis (w_MolDx and wo_MolDx) (*p* < 0.05). **F** Distribution of diagnosed patients according to the IUIS classification

Molecular diagnosis (w_MolDx) was found in 44 patients (44/173, 25.43%) and 129 patients (129/173, 74.57%) remained without genetic diagnosis (wo_MolDx) (Fig. 1A). The diagnostic rate was the same between females and males (22/22). Twenty-five (25/44) and 31 (31/129) patients presented with symptoms before the age of 18 in the w_MolDx and wo_MolDx groups, respectively. These results are consistent with the previously reported [15] higher molecular diagnostic rate in patients with presentation before 18 years of age (*p* < 0.001).

The cohort of patients studied (*n* = 173) was mainly Spaniards (*n* = 157; 90.75%), followed by Latinos (*n* = 13; 7.52%), and others (*n* = 3; 1.73%). Patients with a positive genetic study in our cohort (*n* = 44) were Spaniards (*n* = 39; 88.63%), Latinos (*n* = 3; 6.82%), and other (*n* = 2; 4.55%) (Table S4).

More than one-third (36.36%, 16/44) of the w_MolDx patients were between 18 and 29 years followed by 30 and 39 (22.73%, 10/44) and 41 and 49 (22.73%, 10/44) (Fig. 1B). Related to the clinical phenotype of the patients, among the w_MolDx group, approximately two-thirds of the patients (63.64%, 28/44) suffered infections. It is also interesting that events associated with lymphoproliferation (adenopathies, splenomegaly, and/or hepatomegaly) were present in more than half of the patients (56.82%, 25/44), autoimmunity (thrombocytopenia, autoimmune hemolytic anemia, and/or neutropenia) in more than one-third (40.91%, 18/44), followed by neoplasia (34.09%, 15/44) and hypogammaglobulinemia (27.27%, 12/44) (Fig. 1C). In the wo_MolDx group, the most prominent clinical phenotype was infection (75.19%, 97/129), followed by hypogammaglobulinemia (61.24%, 79/129), pulmonary disorders (44.96%, 58/129), and gastrointestinal disorders (32.56%, 42/129) (Fig. 1D). These data reflect that infection is the main clinical feature in both cohorts, and immune dysregulation, defined as at least one of the following: lymphoproliferation (splenomegaly, hepatomegaly, and lymphadenopathy) and/or autoimmunity (thrombocytopenia, autoimmune hemolytic anemia, and/or neutropenia) [15], was significantly more frequent in the w_MolDx group (65.90%, 29/44) vs. the wo_MolDx group (48.06%, 62/129) (Fig. 1E).

The Jeffrey Model Foundation (JMF) warning signs consist of a list of 10 clinical symptoms related to IEI. This list is available for children or adults and could be a useful tool for physicians, especially for general practitioners, to refer patients to specialized centers for primary immunodeficiency studies (Table S5). Despite the potential utility of JMF warning signs, they are mainly oriented to an infectious perspective and IEI-phenotypes are not only aligned with an increase in infectious susceptibility but also with a growing diversity of symptoms. In addition, approximately one-third of patients with IEI do not meet the criteria from the list [16]. Regarding those limitations and considering the immune dysregulation enrichment in the w_MolDx cohort, we have proposed to include immune dysregulation as a new item of the JMF warning signs (JMF_dys) (Table S6). To check if this new proposal (JMF_dys) is better than the classic JMF warning signs for discovering more IEI patients with molecular diagnosis, we carried out a pairwise comparison of both models as diagnostic tests.

We used the presence or absence of a molecular diagnosis as the gold standard. A total of 95/173 patients (54.91%) fulfilled at least two classic JMF warning signs, 24/44 (54.54%) for the w_MolDx group and 71/129 (55.04%) for the wo_MolDx group. On the other hand, using JMF_dys, 138/173 (79.77%) were positive, 41/44 (93.18%) for w_MolDx and 97/129 (75.19%) for wo_MolDx. After the statistical analysis was performed (Table S7), JMF_dys sensitivity and negative predictive value (NPV) were significantly superior to classic JMF warning signs. In contrast, the specificity decreased significantly when immune dysregulation was used as a new criterion for JMF warning signs. No significant differences were observed for the positive predictive value (PPV). Considering that the JMF warning signs have the purpose of a pseudo-screening test, an improvement in sensitivity would be an interesting property to achieve. Therefore, in view of the results obtained, it could be relevant to add immune dysregulation to the JMF warning signs to improve early IEI diagnosis in adult patients [15–18].

### Molecular Diagnosis Performance of the Requesting Departments

The hospital department requesting the highest number of molecular studies was adult immunology (54.3%, 94/173), followed by the pediatric immunology unit (17.34%, 30/173), internal medicine (13.87%, 24/173), hematology (9.25%, 16/173), dermatology (2.31%, 4/173), and gastrointestinal (1.16%, 2/173) (Fig. S2A). The best diagnostic rate was obtained in this order: hematology (50%, 8/16), gastrointestinal (50%, 1/2, but only 2 patients remitted), pediatric immunology (36.67%, 11/30), dermatology (25.00%, 1/4, but only 4 patients remitted), adult immunology (22.34%, 21/94), and internal medicine (8.33%, 2/24) (Fig. S2B).

### Autoimmune Lymphoproliferative Syndrome (ALPS) was the Most Frequent Genetic Diagnosis in Adult IEI Patients

According to the IUIS classification, diseases of immune dysregulation (group 4) were the most represented category among adult IEI patients (38.64%, 17/44), followed at the same level by predominantly antibody deficiencies (group 3) (13.64%, 6/44) and defects of phagocyte number or function (group 5) (13.64%, 6/44), combined immunodeficiencies (CID) with associated/syndromic features (group 2) (11.36%, 5/44), CID (group 1) (9.09%, 4/44), phenocopies of IEI (group 10) (6.8%, 3/44), autoinflammatory disorders (group 7) (2.27%, 1/44), complement deficiencies (group 8) (2.27%, 1/44), and finally defects in intrinsic and innate immunity (group 6) (2.27%, 1/44) (Fig. 1F). The highest frequency in the molecular diagnosis of immune dysregulation disorders could be explained by the fact that our center specializes in the diagnosis and management of these pathologies, having contributed to the description of new genetic variants [17, 19–21]. On the other hand, no patient was identified as belonging to group 9 (bone marrow failure) (Fig. 1F).

Detailed genetic characteristics of the 44 patients with molecular diagnosis are shown in Table 1 and S8. Eleven patients (25%) with autoimmune lymphoproliferative syndrome (ALPS) due to germline and somatic *FAS* gene variants along with a germline *FASL* gene variant were the most common pathogenic finding [19–22]. We also detected four patients with germline variants in *GATA2* gene (9.1%) and susceptibility to mycobacteria, myelodysplastic syndrome, acute myelogenous leukemia, chronic myelomonocytic leukemia, and/or lymphedema [23]; three patients (6.8%) with variants in *CTLA4* gene and immune dysregulation; three patients (6.8%) with variants in *TET2* gene and ALPS-like phenotype, one of these patients with the biallelic form and two with the monoallelic form of the disease [24]; two related patients (4.5%) with a congenital neutropenia due to an ELANE genetic defect; two unrelated patients with NFKB1 deficiency (4.5%) and common variable immunodeficiency (CVID) phenotype; two brothers (4.5%) were compound heterozygous for variants in *PGM3* gene with very high IgE levels, immunodeficiency, and severe atopy; two unrelated patients (4.5%) with the autosomal dominant (AD) form of activated phosphoinositide 3-kinase syndrome type 2 (APDS2) [25] characterized by severe bacterial infections, reduced memory B cells, and increased transitional B cells, lymphadenopathy/splenomegaly and lymphoproliferation/lymphoma; two brothers with a Griscelli syndrome type 2 due to a *RAB27A* molecular defect (4.5%) with neurological symptoms without albinism and decreased NK cytotoxicity and degranulation; two unrelated patients with Job syndrome and hyper IgE due to *STAT3* AD loss of function (LOF) variants (4.5%); two unrelated patients (4.5%) with a Di George syndrome due to del22q11.2 and immune dysregulation. Finally, single patients were also diagnosed with ADA2, BTK, C8B, CD8A, LIG4, MAGT1, TACI, TAP1, and TLR7 deficiencies.

**Table 1:** Variants detected in adult patients

Patient ID	Gene	Variant (cDNA)	Effect	Zygosity	Variant type	Methodology	Reported	ACMG classification	HSCT (Indication))
IEI-1	*ADA2*	c.1348G > A	p.Gly450Arg	Homozygous	Germline	WES	No^a^	LP	Yes (lymphoma)
IEI-2	*BTK*	c.895-11C > A	Exon 11 deletion	Hemizygous	Germline	Sanger	[26]	LP	No
IEI-3	*C8B*	c.1126C > T	p.Arg376Ter	Compound heterozygous	Germline	Targeted NGS	[27]	Pathogenic	No
		c.205C > T	p,Arg69Ter		Germline	Targeted NGS	[28]	Pathogenic	
IEI-4	*CD8A*	c.272G > A	p.Gly91Asp	Homozygous	Germline	Targeted NGS	No	VUS*	No
IEI-5	*CTLA4*	c.568-2A > G	ND	Heterozygous	Germline	Sanger	No	LP	No
IEI-6	*CTLA4*	c.425G > A	p.Gly142Asp	Heterozygous	Germline	WES	No	LP	No
IEI-7	*CTLA4*	c.253 T > C	p.Cys85Arg	Heterozygous	Germline	Sanger	No^b^	LP	No
IEI-8.1	*ELANE*	c.133G > T	p.Val45Leu	Heterozygous	Germline	Sanger	[29]	LP	No
IEI-8.4	*ELANE*	c.133G > T	p.Val45Leu	Heterozygous	Germline	Sanger	[29]	LP	No
IEI-9.1	*FAS*	c.632dupA	p.Ser212IlefsTer117	Heterozygous	Germline	Sanger	No	LP	No
IEI-9.2	*FAS*	c.632dupA	p.Ser212IlefsTer117	Heterozygous	Germline	Sanger	No	LP	Yes (lymphoma)
IEI-10	*FAS*	c.753dupG	p.Asn252GlufsTer5	Heterozygous	Germline	Sanger	No	LP	No
IEI-11.1	*FAS*	c.905_924dup AAAATTCAAACTTCAGAAAT	p.Glu309LysfsTer38	Heterozygous	Germline	Targeted NGS	[30]	LP	No
IEI-11.2	*FAS*	c.905_924dup AAAATTCAAACTTCAGAAAT	p.Glu309LysfsTer38	Heterozygous	Germline	Sanger	[30]	LP	No
IEI-12	*FAS*	c.686 T > A	p.Leu229Ter	Heterozygous	Somatic	Sanger	[31]	Pathogenic	No
IEI-13	*FAS*	c.749G > A	p.Arg250Gln	Heterozygous	Somatic	Sanger	[31]	LP	No
IEI-14	*FAS*	c.679delG	p.Val227fsTer229	Heterozygous	Somatic	Sanger	[22]	Pathogenic	No
IEI-15	*FAS*	c.979 T > G	p.Ile246Ser	Heterozygous	Germline	Sanger	[32]	Pathogenic	No
IEI-16	*FAS*	c.580delG	p.Glu194LysfsTer22	Heterozygous	Germline	Sanger	[33]	Pathogenic	No
IEI-17	*FASL*	c.740C > A	p.Ala247Glu	Homozygous	Germline	Sanger	[19]	LP	No
IEI-18	*GATA2*	c.913C > G	p.Leu305Val	Heterozygous	Germline	Sanger	No	LP	Yes (MonoMac)
IEI-19	*GATA2*	c.869C > A	p.Ser290Ter	Heterozygous	Germline	Sanger	[34]	Pathogenic	Yes (MonoMac)
IEI-20	*GATA2*	c.708delC	p.Met236IlefsTer325	Heterozygous	Germline	Sanger	[23]	Pathogenic	Yes (MonoMac)
IEI-21	*GATA2*	c.1061C > T	p.Thr354Met	Heterozygous	Germline	Sanger	[23]	LP	No
IEI-22	*LIG4*	c.833G > A	p.Arg278His	Homozygous	Germline	Targeted NGS	[35]	LP	Yes (Infections)
IEI-23	*MAGT1*	c.530delA	p.Asn177IlefsTer4	Hemizygous	Germline	Targeted NGS	No	LP	No
IEI-24	*NFKB1*	c.1597C > T	p.Gln533Ter	Heterozygous	Germline	Targeted NGS	No	LP	No
IEI-25	*NFKB1*	c.1110_1119delTTTTTCGGAT	p.Asn370fsTer470	Heterozygous	Germline	Targeted NGS	No	LP	No
IEI-26.1	*PGM3*	c.1438_1442delTTAAG	p.Leu480SerfsTer10	Compound heterozygous	Germline	Targeted NGS	[36]	Pathogenic	No
		c.1475C > T	p.Thr492Ile		Germline	Targeted NGS	[37]	LP	
IEI-26.3	*PGM3*	c.1438_1442delTTAAG	p.Leu480SerfsTer10	Compound heterozygous	Germline	Targeted NGS	[36]	Pathogenic	No
		c.1475C > T	p.Thr492Ile		Germline	Targeted NGS	[37]	LP	
IEI-27	*PIK3R1*	c.1425 + 1G > T	Exon 11 deletion	Heterozygous	Germline	WES	[25]	Pathogenic	No
IEI-28	*PIK3R1*	c.1425 + 2delT	ND	Heterozygous	Germline	Sanger	[38]	LP	Yes (lymphoma)
IEI-29.1	*RAB27A*	c.227C > T	p.Ala76Val	Homozygous	Germline	Targeted NGS	[39]	LP	No
IEI-29.3	*RAB27A*	c.227C > T	p.Ala76Val	Homozygous	Germline	Targeted NGS	[39]	LP	No
IEI-30	*STAT3 LOF*	c.1311C > A	p.His437Gln	Heterozygous	Germline	Sanger	[40]	LP	No
IEI-31	*STAT3 LOF*	c.1863C > G	p.Phe621Leu	Heterozygous	Germline	Sanger	[41]	Pathogenic	No
IEI-32	*TAP1*	c.2059G > T	p.Glu687Ter	Homozygous	Germline	Targeted NGS	[42]	LP	No
IEI-33	*TBX1*	del22q11.2	ND	Heterozygous	Germline	WES	[43]	Pathogenic	No
IEI-34	*TBX1*	del22q11.2	ND	Heterozygous	Germline	FISH	[43]	Pathogenic	No
IEI-35.1	*TET2*	c.1793delA	p.Asn598IlefsTer3	Compound heterozygous	Germline	WES	[24]	LP	Yes (lymphoma)
		c.277G > T	p.Gly93Ter		Germline	WES	[24]	LP	
IEI-35.3	*TET2*	c.1793delA	p.Asn598IlefsTer3	Heterozygous	Germline	Sanger	[24]	LP	No
IEI-36	*TET2*	del724 Kb	ND	Heterozygous	Germline	CGH Array	[44]	Pathogenic	Yes (lymphoma)
IEI-37	*TLR7*	c.2050A > T	p.Lys684Ter	Hemizygous	Germline	WES	[45]	Pathogenic	No
IEI-38	*TNFRSF13B*	c.198C > A	p.Cys66Ter	Heterozygous	Germline	Targeted NGS	[46]	Pathogenic	No

The American College of Medical Genetics and Genomics (AMCG) classification included an in-house interpretation for some patients; *HSCT*, hematopoietic stem cell transplant; *LP*, likely pathogenic; *ND*, not demonstrated; *VUS*, variant of unknown significance; *a*, the variant was not reported, but c.1348G > T, p.Gly450Cys [47] was described in the same amino acid; *b*, the variant was not reported, but c.254G > A, p.Cys85Tyr [48] was described in the same amino acid. Familial relation for the extra relatives (IEI-N.1 for index case, IEI-N.2 for sister, IEI-N.3 for brother, and IEI-N.4 for cousin). *CD8 deficiency demonstrated by flow cytometry (Fig. S3)

CVID is recognized as the most prevalent IEI in adulthood but a specific genetic cause has only been identified in approximately 20% of patients and the diagnosis is made exclusively following clinical findings [49–51]. Although more than 25 monogenic defects have been described in CVID patients [1, 2], other more complex scenarios are challenging to diagnose in these patients, including epigenetic modifications and somatic variants among others [9]. We obtained a genetic diagnosis in 13.3% of adult patients previously classified as CVID (10/75), where heterozygous variants in *TNFRSF13B* (TACI) were excluded (except for one pathogenic variant), due to the fact that such variants are likely disease modifying rather than disease causing. As expected, the typical clinical phenotype of CVID (infections, hypogammaglobulinemia, and pulmonary disorders) was better represented in the wo_MolDx group (Fig. 1D).

**Fig. 2:**
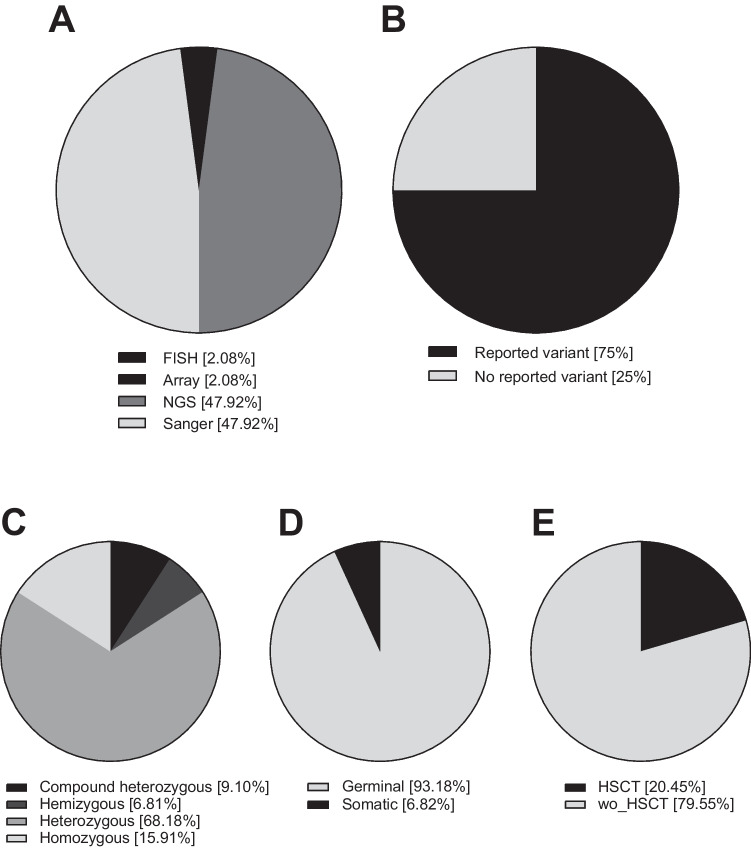
**A** Methods for genetic screening of 48 variants described in 44 patients**. B** Reported vs. no reported molecular variants**. C** Distribution of zygosity for the patients for whom variants were identified in the IEI cohort (*n* = 44 patients). **D** Distribution of patients depending on the type of molecular variant (germline or somatic).** E** Percentage of HSCT patients and without HSCT (wo_HSCT)

A total of 48 variants were described, of which 40 were different. Twenty-three variants (23/48, 47.92%) were identified by Sanger sequencing, most of which occurred between 2005 and 2015. NGS also revealed 23 variants (23/48, 47.92%), either by targeted gene panel (33.33%) or WES (14.58%). CGH array and FISH identified a CNV in the *TET2* gene (2.1%) and a deletion in 22q11.2 (2.1%), respectively (Fig. 2A). More than two-thirds (75%) of the variants had been previously reported; in some cases, they were described by our group for the first time [19, 22–25, 31–33, 38, 45, 52] (Fig. 2B). Most of the patients diagnosed were heterozygous presenting an AD IEI (68.18%), followed by AR inheritance with homozygous (15.91%) or compound heterozygous (9.10%) variants, and finally X-linked with hemizygous variants (6.81%) (Fig. 2C).

It is remarkable that patient IEI-6 was the only case confirmed as a germline de novo variant. The sequencing data from this patient was examined and ruled out for any signs of mosaicism due to its variant allele frequency (VAF) being 53.1%. Germline variants are assumed when VAF is in the range of between 44.1 and 55.6% [53]. In addition, the only potential case of incomplete penetrance that we could confirm was the IEI-7 patient.

### Molecular Performance for the Identification of Somatic Variants Causing IEI-Phenocopies

Somatic mosaicism is described as the presence of a subset of genetically distinct cells due to postzygotic modifications. When a somatic variant produces an IEI-like phenotype, it could be termed a germline IEI mimic variant [54]. There is an increased interest in these type of variants, not only in the pediatric population but also in adults, as somatic defects could explain a considerable number of patients with delayed-onset symptoms [3]. WES is considered a limited technique to correctly identify somatic variants [54]. Extracting DNA from specific immune cellular subpopulations or using targeted high-depth sequencing techniques could be an option to discover somatic variants in both pediatric and adult patients [22]. Following this second methodology, we were able to identify three adult patients with somatic *FAS* variants in our adult cohort (6.8%) (Fig. 2D) [22]. The challenge to identify new somatic variants in adult patients without a germline diagnosis of IEI is still open.

## Hematopoietic stem cell transplantation is a curative therapeutic approach in IEI adult patients

One-fifth of the patients (20.45%, 9/44) in the cohort w_MolDx received allogeneic hematopoietic stem cell transplantation (HSCT) (Fig. 2E). Only the patient with the biallelic form of *TET2* died after HSCT [24]. Although HSCT is considered the main therapeutic and curative approach for children with IEI, it has been relatively unusual among IEI-adults due to difficulties related to the selection of adequate candidates and donors, optimal timing, conditioning regimens, and specific management after transplantation. The decision to transplant remains a complex clinical decision [55]. Nevertheless, negative HSCT outcomes have been mostly related to complications arising from the specific IEI of the patient, more than with the age of the candidates [56]. Therefore, an early molecular diagnosis is imperative to allow IEI adults to benefit from HSCT and avoid the occurrence of adverse effects that would contraindicate it.

## Discussion

The aim of this work is to show that IEI-related genetic alterations are not infrequent findings in adult patients. As a rule, clinical manifestations due to genetic alterations usually appear in childhood and suspicion of IEI-causing variants in adult patients is less frequent. The delay from initial manifestations to diagnosis of IEI for index cases frequently takes several years. Sometimes, this molecular diagnostic delay can be critical and often involves frequent, costly, and unhelpful specialist visits and unnecessary laboratory tests, which represents a diagnostic odyssey. It should be necessary to expand access to screening of adult patients for IEI, especially when disease onset occurs in childhood [8]. In this work, patients with initial symptoms before 18 years of age should have been studied as soon as possible, but several reason could have influenced the delay of a molecular diagnosis: a. symptomatic delay of the disease with a poor specificity of presenting symptoms, as in the case of some CVID patients; b. multisystemic manifestations without a multidisciplinary evaluation (TET2 deficiency, STAT3 LoF, 22q11.2del patients); c. limited access to NGS techniques in the healthcare field when the patients were under 18 years of age (PGM3, PIK3R1, RAB27A patients); d. incomplete penetrance with mild clinical phenotypes where the diagnosis was carried out through the patients’ offspring (ELANE patients).

Unfortunately, the resulting diagnostic delay can interfere with properly tailored treatment, causing unnecessary distress, diminished quality of life, and sometimes death. A point to take into account is the case of patients with CVID, where approximately 50% of the patients’ onset is in the second decade of life [15], and in some cases, molecular diagnosis represents a challenge.

In conclusion, NGS is a first-tier diagnostic test for monogenic adult IEI that can be used in patients with typical and atypical presentation. Therefore, adult patients with immune dysregulation in the form of autoimmunity, cytopenias, lymphoproliferation, and neoplasia should be considered for NGS screening.

The ever-changing clinical and genetic landscape of IEI and the advances of the last decade have shown how clinical, translational, and basic science networks have enhanced the field of immunodeficiencies [57]. The growing number of genetic discoveries has increased clinical awareness, and targeted biologics and definitive treatments such as gene therapy will be important in the next decade.

### Supplementary Information

Below is the link to the electronic supplementary material.

Supplementary file1 (DOCX 1169 KB)

## Data Availability

The datasets generated during and/or analyzed during the current study are available from the corresponding author upon reasonable request.

## Abbreviations

ALPS: Autoimmune lymphoproliferative syndrome
IEI: Inborn errors of immunity
IUIS: International Union of Immunological Societies Expert Committee
NGS: Next generation sequencing
WES: Whole exome sequencing

## References

[1] Bousfiha A, Moundir A, Tangye SG, Picard C, Jeddane L, Al-Herz W (2022). The 2022 Update of IUIS Phenotypical Classification for Human Inborn Errors of Immunity. J Clin Immunol..

[2] Tangye SG, Al-Herz W, Bousfiha A, Cunningham-Rundles C, Franco JL, Holland SM, et al. (2022) Human Inborn Errors of Immunity: 2022 Update on the Classification from the International Union of Immunological Societies Expert Committee 42 Journal of Clinical Immunology. Springer US 1473–1507 Available from: 10.1007/s10875-022-01289-3 10.1007/s10875-022-01289-3 PMC9244088 35748970

[3] Staels F, Collignon T, Betrains A, Gerbaux M, Willemsen M, Humblet-Baron S (2021). Monogenic adult-onset inborn errors of immunity. Front Immunol.

[4] Stoddard JL, Niemela JE, Fleisher TA, Rosenzweig SD (2014). Targeted NGS: A cost-effective approach to molecular diagnosis of PIDs. Front Immunol.

[5] Platt CD, Zaman F, Bainter W, Stafstrom K, Almutairi A, Reigle M (2021). Efficacy and economics of targeted panel versus whole-exome sequencing in 878 patients with suspected primary immunodeficiency. J Allergy Clin Immunol.

[6] Al-Mousa H, Abouelhoda M, Monies DM, Al-Tassan N, Al-Ghonaium A, Al-Saud B (2016). Unbiased targeted next-generation sequencing molecular approach for primary immunodeficiency diseases. J Allergy Clin Immunol.

[7] Stray-Pedersen A, Sorte HS, Samarakoon P, Gambin T, Chinn IK, Coban Akdemir ZH (2017). Primary immunodeficiency diseases: genomic approaches delineate heterogeneous Mendelian disorders. J Allergy Clin Immunol.

[8] Quinn J, Modell V, Johnson B, Poll S, Aradhya S, Orange JS, et al. Global expansion of Jeffrey’s Insights: Jeffrey Modell Foundation’s genetic sequencing program for primary immunodeficiency. Front Immunol. 2022;13:906540. 10.3389/fimmu.2022.906540 PMC9226364 35757720

[9] de Valles-Ibáñez G, Esteve-Solé A, Piquer M, Azucena González-Navarro E, Hernandez-Rodriguez J, Laayouni H, et al. Evaluating the genetics of common variable immunodeficiency: monogenetic model and beyond. Front Immunol. 2018;9:636. 10.3389/fimmu.2018.00636 PMC5960686 29867916

[10] Roy S, Coldren C, Karunamurthy A, Kip NS, Klee EW, Lincoln SE (2018). Standards and guidelines for validating next-generation sequencing bioinformatics pipelines: a joint recommendation of the Association for Molecular Pathology and the College of American Pathologists. J Mol Diagn.

[11] Karczewski KJ, Francioli LC, Tiao G, Cummings BB, Alföldi J, Wang Q (2020). The mutational constraint spectrum quantified from variation in 141,456 humans. Nature.

[12] Richards S, Aziz N, Bale S, Bick D, Das S, Gastier-Foster J (2015). Standards and guidelines for the interpretation of sequence variants: a joint consensus recommendation of the American College of Medical Genetics and Genomics and the Association for Molecular Pathology. Genet Med.

[13] Kopanos C, Tsiolkas V, Kouris A, Chapple CE, Albarca Aguilera M, Meyer R (2019). VarSome: the human genomic variant search engine. Bioinformatics.

[14] Kosinski AS (2013). A weighted generalized score statistic for comparison of predictive values of diagnostic tests. Stat Med.

[15] Thalhammer J, Kindle G, Nieters A, Rusch S, Seppänen MRJ, Fischer A (2021). Initial presenting manifestations in 16,486 patients with inborn errors of immunity include infections and noninfectious manifestations. J Allergy Clin Immunol.

[16] Dąbrowska A, Grześk E, Urbańczyk A, Mazalon M, Grześk G, Styczyński J, et al. Extended list of warning signs in qualification to diagnosis and treatment of inborn errors of immunity in children and young adults. J Clin Med. 2023;12(10):3401. 10.3390/jcm12103401 PMC10219467 37240507

[17] López-Nevado M, González-Granado LI, Ruiz-García R, Pleguezuelo D, Cabrera-Marante O, Salmón N, et al. Primary immune regulatory disorders with an autoimmune lymphoproliferative syndrome-like phenotype: immunologic evaluation, early diagnosis and management. Front Immunol. 2021;12:671755. 10.3389/fimmu.2021.671755 PMC8382720 34447369

[18] Schiavo E, Martini B, Attardi E, Consonni F, Ciullini Mannurita S, Coniglio ML, et al. Autoimmune cytopenias and dysregulated immunophenotype act as warning signs of inborn errors of immunity: results from a prospective study. Front Immunol. 2022;12:790455. 10.3389/fimmu.2021.790455 PMC8765341 35058929

[19] Del-Rey M, Ruiz-Contreras J, Bosque A, Calleja S, Gomez-Rial J, Roldan E (2006). A homozygous Fas ligand gene mutation in a patient causes a new type of autoimmune lymphoproliferative syndrome. Blood.

[20] Ruiz-Garcia R, Mora S, Lozano-Sanchez G, Martinez-Lostao L, Paz-Artal E, Ruiz-Contreras J (2015). Decreased activation-induced cell death by EBV-transformed B-cells from a patient with autoimmune lymphoproliferative syndrome caused by a novel FASLG mutation. Pediatr Res.

[21] Casamayor-Polo L, López-Nevado M, Paz-Artal E, Anel A, Rieux-Laucat F, Allende LM. Immunologic evaluation and genetic defects of apoptosis in patients with autoimmune lymphoproliferative syndrome (ALPS). Crit Rev Clin Lab Sci. 2021;58(4):253–74. 10.1080/10408363.2020.1855623 33356695

[22] López-Nevado M, Docampo-Cordeiro J, Ramos JT, Rodríguez-Pena R, Gil-López C, Sánchez-Ramón S, et al. Next generation sequencing for detecting somatic fas mutations in patients with autoimmune lymphoproliferative syndrome. Front Immunol. 2021;12:656356. 10.3389/fimmu.2021.656356 PMC8117005 33995372

[23] Ruiz-García R, Rodríguez-Vigil C, Marco FM, Gallego-Bustos F, Castro-Panete MJ, Diez-Alonso L (2017). Acquired senescent T-cell phenotype correlates with clinical severity in GATA binding protein 2-deficient patients. Front Immunol..

[24] López-Nevado M, Ortiz-Martín J, Serrano C, Pérez-Saez MA, López-Lorenzo JL, Gil-Etayo FJ (2023). Novel germline TET2 mutations in two unrelated patients with autoimmune lymphoproliferative syndrome-like phenotype and hematologic malignancy. J Clin Immunol.

[25] Dominguez-Pinilla N, Allende LM, Rosain J, del Carmen Gallego M, Chaves F, Deswarte C (2018). Disseminated abscesses due to Mycoplasma faucium in a patient with activated PI3Kδ syndrome type 2. J allergy Clin Immunol Pract..

[26] Maekawa K, Yamada M, Okura Y, Sato Y, Yamada Y, Kawamura N (2010). X-linked agammaglobulinemia in a 10-year-old boy with a novel non-invariant splice-site mutation in Btk gene. Blood Cells Mol Dis.

[27] Dellepiane RM, Dell’Era L, Pavesi P, Macor P, Giordano M, De Maso L, et al. Invasive meningococcal disease in three siblings with hereditary deficiency of the 8(th) component of complement: evidence for the importance of an early diagnosis. Orphanet J Rare Dis. 2016;11(1):64. 10.1186/s13023-016-0448-5 PMC4869260 27183977

[28] Saucedo L, Ackermann L, Platonov AE, Gewurz A, Rakita RM, Densen P (1995). Delineation of additional genetic bases for C8 beta deficiency. Prevalence of null alleles and predominance of C-->T transition in their genesis. J Immunol.

[29] Germeshausen M, Deerberg S, Peter Y, Reimer C, Kratz CP, Ballmaier M (2013). The spectrum of ELANE mutations and their implications in severe congenital and cyclic neutropenia. Hum Mutat.

[30] Rieux-Laucat F, Le Deist F, Hivroz C, Roberts IAG, Debatin KM, Fischer A (1995). Mutations in fas associated with human lymphoproliferative syndrome and autoimmunity. Science..

[31] Martínez-Feito A, Melero J, Mora-Díaz S, Rodríguez-Vigil C, Elduayen R, González-Granado LI (2016). Autoimmune lymphoproliferative syndrome due to somatic FAS mutation (ALPS-sFAS) combined with a germline caspase-10 (CASP10) variation. Immunobiology.

[32] Del-Rey MJ, Manzanares J, Bosque A, Aguiló JI, Gómez-Rial J, Roldan E (2007). Autoimmune lymphoproliferative syndrome (ALPS) in a patient with a new germline Fas gene mutation. Immunobiology.

[33] Bilbao Aburto A, Arana Aguirre N, García MartNez JM, Astigarraga Aguirre I, Allende LM (2010). Familial splenomegaly as a first clinical sign of autoimmune lymphoproliferative syndrome. An Pediatr (Barc).

[34] Azevedo L, Jay A, Lorenzana A, Keel S, Abraham RS, Horwitz M, et al. Case Report of an adolescent male with unexplained pancytopenia: GATA2-associated bone marrow failure and genetic testing. Glob Pediatr Health. 2017;4:2333794X17744947. 10.1177/2333794X17744947 PMC5718303 29230432

[35] O’Driscoll M, Cerosaletti KM, Girard PM, Dai Y, Stumm M, Kysela B (2001). DNA ligase IV mutations identified in patients exhibiting developmental delay and immunodeficiency. Mol Cell.

[36] Zhang Y, Yu X, Ichikawa M, Lyons JJ, Datta S, Lamborn IT, et al. Autosomal recessive phosphoglucomutase 3 (PGM3) mutations link glycosylation defects to atopy, immune deficiency, autoimmunity, and neurocognitive impairment. J Allergy Clin Immunol. 2014;133(5):1400–9. 10.1016/j.jaci.2014.02.013 PMC4016982 24589341

[37] García-García A, Buendia Arellano M, Deyà-Martínez À, Lozano Blasco J, Serrano M, Van Den Rym A (2021). Novel PGM3 compound heterozygous variants with IgE-related dermatitis, lymphopenia, without syndromic features. Pediatr Allergy Immunol.

[38] Inglés-Ferrándiz M, Martin-Inaraja M, Herrera L, Villaverde M, Santos S, Vesga MA, et al. Generation, establishment and characterization of a pluripotent stem cell line (CVTTHi001-A) from primary fibroblasts isolated from a patient with activated PI3 kinase delta syndrome (APDS2). Stem Cell Res. 2020;49:102082. 10.1016/j.scr.2020.102082 33221676

[39] Cetica V, Hackmann Y, Grieve S, Sieni E, Ciambotti B, Coniglio ML (2015). Patients with Griscelli syndrome and normal pigmentation identify RAB27A mutations that selectively disrupt MUNC13-4 binding. J Allergy Clin Immunol.

[40] Minegishi Y, Saito M, Tsuchiya S, Tsuge I, Takada H, Hara T (2007). Dominant-negative mutations in the DNA-binding domain of STAT3 cause hyper-IgE syndrome. Nature.

[41] Kumánovics A, Wittwer CT, Pryor RJ, Augustine NH, Leppert MF, Carey JC (2010). Rapid molecular analysis of the STAT3 gene in Job syndrome of hyper-IgE and recurrent infectious diseases. J Mol Diagn.

[42] Villa-Forte A, De La Salle H, Fricker D, Hentges F, Zimmer J (2008). HLA class I deficiency syndrome mimicking Wegener’s granulomatosis. Arthritis Rheum.

[43] Adachi M, Tachibana K, Masuno M, Makita Y, Maesaka H, Okada T, et al. Clinical characteristics of children with hypoparathyroidism due to 22q11.2 microdeletion. Eur J Pediatr. 1998;157(1):34–8. 10.1007/s004310050762 9461360

[44] Viguié F, Aboura A, Bouscary D, Ramond S, Delmer A, Tachdjian G (2005). Common 4q24 deletion in four cases of hematopoietic malignancy: early stem cell involvement?. Leukemia.

[45] Asano T, Boisson B, Onodi F, Matuozzo D, Moncada-Velez M, Renkilaraj MRLM, et al. X-linked recessive TLR7 deficiency in ~1% of men under 60 years old with life-threatening COVID-19. Sci Immunol. 2021;6(62):eabl4348. 10.1126/sciimmunol.abl4348 PMC8532080 34413140

[46] Salzer U, Chapel HM, Webster ADB, Pan-Hammarström Q, Schmitt-Graeff A, Schlesier M (2005). Mutations in TNFRSF13B encoding TACI are associated with common variable immunodeficiency in humans. Nat Genet.

[47] Rama M, Duflos C, Melki I, Bessis D, Bonhomme A, Martin H (2018). A decision tree for the genetic diagnosis of deficiency of adenosine deaminase 2 (DADA2): a French reference centres experience. Eur J Hum Genet.

[48] Schwab C, Gabrysch A, Olbrich P, Patiño V, Warnatz K, Wolff D (2018). Phenotype, penetrance, and treatment of 133 cytotoxic T-lymphocyte antigen 4–insufficient subjects. J Allergy Clin Immunol.

[49] Mormile I, Punziano A, Riolo CA, Granata F, Williams M, de Paulis A (2021). Common variable immunodeficiency and autoimmune diseases: a retrospective study of 95 adult patients in a single tertiary care center. Front Immunol.

[50] Ramirez NJ, Posadas-Cantera S, Caballero-Oteyza A, Camacho-Ordonez N, Grimbacher B (2021). There is no gene for CVID — novel monogenetic causes for primary antibody deficiency. Curr Opin Immunol.

[51] Rojas-Restrepo J, Caballero-Oteyza A, Huebscher K, Haberstroh H, Fliegauf M, Keller B, et al. Establishing the molecular diagnoses in a cohort of 291 patients with predominantly antibody deficiency by targeted next-generation sequencing: experience from a monocentric study. Front Immunol. 2021;12:786516. 10.3389/fimmu.2021.786516 PMC8718408 34975878

[52] Gonzalez-Granado LI, Ruiz-García R, Blas-Espada J, Moreno-Villares JM, Germán-Diaz M, López-Nevado M, et al. Acquired and innate immunity impairment and severe disseminated mycobacterium genavense infection in a patient with a NF-κB1 deficiency. Front Immunol. 2019;9:3148. 10.3389/fimmu.2018.03148 PMC6362422 30761159

[53] Mensa-Vilaró A, Bravo García-Morato M, de la Calle-Martin O, Franco-Jarava C, Martínez-Saavedra MT, González-Granado LI (2019). Unexpected relevant role of gene mosaicism in patients with primary immunodeficiency diseases. J Allergy Clin Immunol.

[54] Aluri J, Cooper MA. Somatic mosaicism in inborn errors of immunity: Current knowledge, challenges, and future perspectives. Semin Immunol. 2023;67:101761. 10.1016/j.smim.2023.101761 PMC11321052 37062181

[55] Morris EC (2020). Allogeneic hematopoietic stem cell transplantation in adults with primary immunodeficiency. Hematol Am Soc Hematol Educ Progr.

[56] Albert MH, Sirait T, Eikema DJ, Bakunina K, Wehr C, Suarez F (2022). Hematopoietic stem cell transplantation for adolescents and adults with inborn errors of immunity: an EBMT IEWP study. Blood.

[57] Walter JE, Ziegler JB, Ballow M, Cunningham-Rundles C (2023). Advances and challenges of the decade: the ever-changing clinical and genetic landscape of immunodeficiency. J allergy Clin Immunol Pract.

