# Supplementary material for: Enrichment of Immune Dysregulation Disorders in Adult Patients with Human Inborn Errors of Immunity
Source: J Clin Immunol. 2024 Feb 16;44(3):61. doi: 10.1007/s10875-024-01664-2 (PMC10873437; doi:10.1007/s10875-024-01664-2)
Supplement: Supplementary file 1 — Supplementary file1 (DOCX 1169 KB) [file 10875_2024_1664_MOESM1_ESM.docx]

**SUPPLEMENTARY DOCUMENTS**

**Supplementary Table 1. Targeted gene-panel involved in IEI (192 genes).**

ACT1, ADA, AICDA, AIRE, AK2, AP3B1, AP3D1, ATM, BCL10, BLNK, BTK, C3, CARD11, CARD9, CASP10, CASP8, CD127, CD19, CD20, CD21, CD27, CD3D, CD3E, CD3G, CD3Z, CD45, CD79A, CD79B, CD81, CD8A, CEBPE, CECR1, Cernunnos, CLEC7A, COPA, CORO1A, CTLA4, CTPS1, CTSC, CXCR4, CYBA, CYBB, DCLRE1C, DKC1, DNMT3B, DOCK2, DOCK8, ELANE, EVER1, EVER2, FADD, FCGR3A, FOXN1, FOXP3, G6PC3, GATA2, GFI1, HAX1, HOIL1, ICOS, IFNGR1, IFNGR2, IGHM, IGLL1, IKZF1, IKBA, IKBKB, IKBKG, IL10, IL10RA, IL10RB, IL12B, IL12RB1, IL12RB2, IL17F, IL17RA, IL17RC, IL1RN, IL21, IL21R, IL2RA, IL2RG, IL7, IRAK4, IRF3, IRF7, IRF8, ISG15, ITGB2, ITK, JAGN1, JAK3, KIND3, KRAS, LAMTOR2, LCK, LIG4, LPIN2, LRBA, LYST, MAGT1, MALT1, MAP3K14, MCM4, MEFV, MHC2TA, MRE11, MST1, MVK, MYD88, NCF1, NCF2, NFKB1, NFKBIA, NFKB2, NHP2, NLRC4, NLRP12, NLRP3, NOD2, NOP10, NRAS, ORAI1, p40phox, PGM3, PIK3CD, PIK3R1, PLCG2, PMS2, PNP, POLE1, PRF1, PRKCD, PSMB8, PSTPIP1, PTPN6, RAB27A, RAG1, RAG2, RFX5, RFXANK, RFXAP, RLTPR, RMRP, RNF168, RORC, RTEL1, SH2D1A, SMARCAL1, SP110, SPINK5, STAT1, STAT2, STAT3, STAT5B, STIM1, STX11, STXBP2, TAP1, TAP2, TAPBP, TBK1, TCF3, TCN2, TERC, TERT, TINF2, TIRAP, TLR3, TMEM173, TNFRSF13B, TNFRSF13C, TNFRSF1A, TNFRSF5, TNFRSF6, TNFSF5, TNFSF6, TRAF3, TRIF, TRNT1, TTC7A, TWEAK, UNC119, UNC13D, UNC93B1, UNG, VPS45, WAS, WIPF1, XIAP, XRCC4, ZAP70, ZBTB24**.**

**Supplementary Table 2. Targeted gene-panel involved in IEI (434 genes):** Highlighted in bold show genes not included in WES.

ACD, ACP5, ACTB, ADA, ADA2, ADAM17, ADAR, AICDA, AIRE, AK2, ALPI, AP1S3, AP3B1, AP3D1, APOL1, ARHGEF1, ARPC1B, **ATG16L1**, ATM, ATP6AP1, B2M, BACH2, BCL10, BCL11B, **BLK**, BLM, BLNK, BTK, C1QA, C1QB, C1QC, C1R, C1S, C2, C2ORF69, C3, C4B, C4B,C4A, C5, C6, C7, C8A, C8B, C8G, C9, CARD11, CARD14, **CARD8**, CARD9, CARMIL2, CARMIL2,ACD, CASP10, CASP8, CCBE1, CD19, CD247, CD27, CD28, CD3D, CD3E, CD3G, CD40, CD40LG, CD46, CD55, CD59, CD70, CD79A, CD79B, CD81, CD8A, CDC42, CDCA7, CEBPE, CFB, CFD, CFH, CFHR1, CFHR2, CFHR3, CFHR4, CFHR5, CFI, CFP, CFTR, CHD7, chr12:7052979-7053041, CIB1, CIITA, CLCN7, CLPB, COPA, CORO1A, CR2, CSF2RA, CSF2RB, CSF3R, CTC1, CTLA4, CTNNBL1, CTPS1, CTSC, CXCR4, CYBA, CYBB, CYBC1, DBR1, DCLRE1C, **DDX58**, DEF6, DKC1, DNAJC21, DNASE1L3, DNASE2, DNMT3B, DOCK2, DOCK8, EFL1, ELANE, EPG5, ERBIN, ERCC6L2, EXTL3, FAAP24, FADD, FAS, FASLG, FAT4, FCGR3A, FCHO1, FCN3, FERMT1, FERMT3, FNIP1, FOXN1, FOXP3, FPR1, G6PC3, G6PD, GATA2, GFI1, GINS1, HAVCR2, HAX1, HELLS, HMOX1, HYOU1, ICOS, ICOSLG, IFIH1, IFNAR1, IFNAR2, IFNG, IFNGR1, IFNGR2, **IGHM**, **IGKC**, IGLL1, IKBKB, IKBKG, IKZF1, IKZF3, IL10, IL10RA, IL10RB, IL12B, IL12RB1, IL12RB2, IL17F, IL17RA, IL17RC, IL18BP, IL1RN, IL21, IL21R, IL23R, IL2RA, IL2RB, IL2RG, IL36RN, IL6R, IL6ST, **IL7**, IL7R, INO80, IRAK1, IRAK4, IRF2BP2, IRF3, IRF4, IRF7, IRF8, IRF9, **IRGM**, ISG15, ITCH, **ITGAM**, ITGB2, ITK, ITPKB, ITPR3, JAGN1, JAK1, JAK3, KDM6A, KMT2A, KMT2D, **KRAS**, LAMTOR2, LAT, LCK, LCP2, LIG1, LIG4, LPIN2, LRBA, **LRRC8A**, LSM11, **LYN**, LYST, MAGT1, MALT1, MAN2B2, MAP1LC3B2, MAP3K14, MAPK8, MASP2, MCM10, MCM4, MEFV, MOGS, MS4A1, MSH6, MSN, MTHFD1, MVK, MYD88, MYSM1, NBAS, NBN, NCF1, NCF2, NCF4, NCKAP1L, NCSTN, NFAT5, NFE2L2, NFKB1, NFKB2, NFKBIA, NHEJ1, NHP2, NLRC4, NLRP1, NLRP12, NLRP3, **NLRP7**, NOD2, NOP10, NOS2, **NRAS**, NSMCE3, OAS1, ORAI1, OSTM1, OTULIN, PARN, PAX1, PDCD1, PEPD, PGM3, PIK3CD, PIK3CG, PIK3R1, PLCG2, PLEKHM1, PMS2, PNP, POLA1, POLD1, POLD2, **POLD3**, **POLD4**, POLE, POLE2, POLR3A, POLR3C, POLR3F, **POMP**, POU2AF1, PRF1, PRKCD, PRKDC, PSEN1, PSENEN, **PSMA3**, **PSMB4**, PSMB8, PSMG2, PSTPIP1, PTEN, PTPRC, RAB27A, RAC2, RAG1, RAG2, RANBP2, RASGRP1, RBCK1, REL, RELA, RELB, RFX5, RFXANK, RFXAP, RHOG, RHOH, RIPK1, RMRP, RNASEH2A, RNASEH2B, RNASEH2C, **RNF113A**, RNF168, RNF31, **RNU4ATAC**, RORC, RPSA, RTEL1, **SAA1**, SAMD9L, SAMHD1, SASH3, SBDS, SEC61A1, SEMA3E, SERPING1, SH2D1A, SH3BP2, SH3KBP1, SKIV2L, SLC29A3, SLC35C1, SLC37A4, SLC39A7, SLC46A1, SLC7A7, SMARCAL1, SMARCD2, SNX10, SOCS1, SP110, SPINK5, SPPL2A, SRP54, SRP72, STAT1, STAT2, STAT3, **STAT4**, STAT5B, STIM1, STK4, STN1, STX11, **STX3**, STXBP2, TAP1, TAP2, TAPBP, TAZ, TBK1, TBX1, TBX21, TCF3, TCIRG1, TCN2, TERC, TERT, TET2, TFRC, TGFB1, TGFBR1, TGFBR2, THBD, TICAM1, TINF2, TIRAP, TLR3, TLR7, TLR8, TMC6, TMC8, TMEM173, TNFAIP3, TNFRSF11A, TNFRSF13B, TNFRSF13C, TNFRSF1A, TNFRSF4, TNFRSF6B, TNFRSF9, TNFSF11, TNFSF12, TNFSF13, **TONSL**, TOP2B, TP53, TPP2, **TPT1**, **TRAC**, TRAF3, TRAF3IP2, TRIM22, TRNT1, TTC37, TTC7A, TYK2, **UBA1**, UNC13D, UNC93B1, UNG, USB1, USP18, **VAV1**, VPS13B, VPS45, WAS, WDR1, WIPF1, WRAP53, XIAP, ZAP70, ZBTB24, ZNF341, ZNFX1.

**Supplementary Table 3. WES filtered by 455 genes involved in IEI.** Highlighted in bold show genes not included in Targeted gene-panel (434 genes):

ACD, ACP5, ACTB, ADA, ADA2, ADAM17, ADAR, AICDA, AIRE, AK2, ALPI, AP1S3, AP3B1, AP3D1, APOL1, ARHGEF1, ARPC1B, **ARPC5**, **ATAD3A**, **ATG4A**, ATM, ATP6AP1, B2M, BACH2, BCL10, BCL11B, BLM, BLNK, **BRCA1**, **BRCA2**, **BRIP1**, BTK, C1QA, C1QB, C1QC, C1R, C1S, C2, C2ORF69, C3, C4A, C4B, C5, C6, C7, C8A, C8B, C8G, C9, CARD11, CARD14, CARD9, CARMIL2, CASP10, CASP8, CCBE1, CD19, CD247, CD27, CD28, CD3D, CD3E, CD3G, CD40, CD40LG, CD46, CD55, CD59, CD70, CD79A, CD79B, CD81, CD8A, CDC42, CDCA7, CEBPE, CFB, CFD, CFH, CFHR1, CFHR2, CFHR3, CFHR4, CFHR5, CFI, CFP, CFTR, CHD7, **CHUK**, CIB1, CIITA, CLCN7, CLPB, COPA, **COPG1**, CORO1A, CR2, **CRACR2A**, CSF2RA, CSF2RB, CSF3R, CTC1, CTLA4, CTNNBL1, CTPS1, CTSC, **CXCR2**, CXCR4, CYBA, CYBB, CYBC1, **DBF4**, DBR1, DCLRE1C, **DDX3X**, DEF6, **DIAPH1**, DKC1, DNAJC21, DNASE1L3, DNASE2, DNMT3B, DOCK2, DOCK8, **DPP9**, EFL1, ELANE, **ELF4**, **ELMO1**, EPG5, ERBIN, **ERCC4**, ERCC6L2, EXTL3, **EZR**, FAAP24, FADD, **FANCA**, **FANCB**, **FANCC**, **FANCD2**, **FANCE**, **FANCF**, **FANCG**, **FANCI**, **FANCL**, **FANCM**, FAS, FASLG, FAT4, FCGR3A, FCHO1, FCN3, FERMT1, FERMT3, FNIP1, **FOXI3**, FOXN1, FOXP3, FPR1, G6PC3, G6PD, GATA2, GFI1, GINS1, HAVCR2, HAX1, **HCK**, HELLS, HMOX1, HYOU1, ICOS, ICOSLG, IFIH1, IFNAR1, IFNAR2, IFNG, IFNGR1, IFNGR2, IGLL1, IKBKB, IKBKG, IKZF1, **IKZF2**, IKZF3, IL10, IL10RA, IL10RB, IL12B, IL12RB1, IL12RB2, IL17F, IL17RA, IL17RC, IL18BP, IL1RN, IL21, IL21R, IL23R, IL2RA, IL2RB, IL2RG, IL36RN, IL6R, IL6ST, IL7R, INO80, IRAK1, IRAK4, IRF2BP2, IRF3, IRF4, IRF7, IRF8, IRF9, ISG15, ITCH, ITGB2, ITK, ITPKB, ITPR3, JAGN1, JAK1, JAK3, KDM6A, KMT2A, KMT2D, LAMTOR2, LAT, LCK, LCP2, LIG1, LIG4, LPIN2, LRBA, **LRRC32**, LSM11, LYST, **MAD2L2**, MAGT1, MALT1, MAN2B2, MAP1LC3B2, MAP3K14, MAPK8, MASP2, MCM10, MCM4, **MECOM**, MEFV, MOGS, **MRTFA**, MS4A1, MSH6, MSN, **MST1R**, MTHFD1, MVK, MYD88, MYSM1, NBAS, NBN, NCF1, NCF2, NCF4, NCKAP1L, NCSTN, NFAT5, **NFATC2**, NFE2L2, NFKB1, NFKB2, NFKBIA, NHEJ1, NHP2, NLRC4, NLRP1, NLRP12, NLRP3, NOD2, NOP10, NOS2, NSMCE3, OAS1, ORAI1, OSTM1, OTULIN, **PALB2**, PARN, PAX1, PDCD1, PEPD, PGM3, PIK3CD, PIK3CG, PIK3R1, PLCG2, PLEKHM1, PMS2, PNP, POLA1, POLD1, POLD2, POLE, POLE2, POLR3A, POLR3C, POLR3F, POU2AF1, PRF1, PRKCD, PRKDC, PSEN1, PSENEN, PSMB8, **PSMB9**, PSMG2, PSTPIP1, PTEN, PTPRC, RAB27A, RAC2, **RAD51**, **RAD51C**, RAG1, RAG2, RANBP2, **RAP1B**, RASGRP1, RBCK1, REL, RELA, RELB, **RFWD3**, RFX5, RFXANK, RFXAP, RHOG, RHOH, RIPK1, RMRP, RNASEH2A, RNASEH2B, RNASEH2C, RNF168, RNF31, RORC, RPSA, RTEL1, **SAMD9**, SAMD9L, SAMHD1, SASH3, SBDS, SEC61A1, SEMA3E, SERPING1, SH2D1A, SH3BP2, SH3KBP1, SKIC2, SKIC3, SLC29A3, SLC35C1, SLC37A4, SLC39A7, SLC46A1, SLC7A7, **SLX4**, SMARCAL1, SMARCD2, SNX10, SOCS1, SP110, **SPI1**, SPINK5, SPPL2A, SRP54, SRP72, STAT1, STAT2, STAT3, STAT5B, STIM1, STING1, STK4, STN1, STX11, STXBP2, **SYK**, TAFAZZIN, TAP1, TAP2, TAPBP, TBK1, TBX1, TBX21, TCF3, TCIRG1, TCN2, TERC, TERT, TET2, TFRC, TGFB1, TGFBR1, TGFBR2, THBD, TICAM1, TINF2, TIRAP, TLR3, TLR7, TLR8, TMC6, TMC8, TNFAIP3, TNFRSF11A, TNFRSF13B, TNFRSF13C, TNFRSF1A, TNFRSF4, TNFRSF6B, TNFRSF9, TNFSF11, TNFSF12, TNFSF13, TOP2B, TP53, TPP2, TRAF3, TRAF3IP2, **TREX1**, TRIM22, TRNT1, TTC7A, TYK2, **UBE2T**, UNC13D, UNC93B1, UNG, USB1, USP18, VPS13B, VPS45, WAS, WDR1, WIPF1, WRAP53, XIAP, **XRCC2**, ZAP70, ZBTB24, ZNF341, ZNFX1.

**Supplementary Table 4. Demographic data.**

|  | |  | | **w_MolDx**  (n=44) | **wo_MolDx**  (n=129) | **Total**  (n=173) |
| --- | --- | --- | --- | --- | --- | --- |
| **Age** | |  | |  |  |  |
|  | Mean (SD) | | | 42.32 (13.76) | 47.18 (14.37) | 45.94 (13.34) |
|  | Median | | | 43.50 | 47.00 | 45.00 |
|  | Range | | | 19-85 | 19-80 | 19-85 |
|  |  | | |  |  |  |
| **Gender, n (%)** | |  | |  |  |  |
|  | Female | | | 22 | 72 (55.81) | 94 (54.34) |
|  | Male | | | 22 | 57 (44.19) | 79 (45.66) |
|  | Female/Male | | | 1.00 | 1.26 | 1.19 |
|  |  | | |  |  |  |
| **Ethnicity, n (%)** | | |  |  |  |  |
|  | Spaniard | | | 39 (88.63) | 118 (91.47) | 157 (90.75) |
|  | Latino | | | 3 (6.82) | 10 (7.75) | 13 (7.52) |
|  | Other | | | 2 (4.55) | 1 (0.78) | 3 (1.73) |

**Supplementary Table 5. The 10 JMF warning signs for adults IEI.**

| **Warning signs in adults** | |
| --- | --- |
| 1. | ≥ 2 new ear infections within 1 year |
| 2. | ≥ 2 new sinus infections within 1 year, in the absence of allergy |
| 3. | 1 pneumonia per year for > 1 year |
| 4. | Chronic diarrhea with weight loss |
| 5. | Recurrent viral infections (colds, herpes, warts, condyloma) |
| 6. | Recurrent need for IV antibiotics to clear infections |
| 7. | Recurrent, deep abscesses of the skin or internal organs |
| 8. | Persistent thrush or fungal infection on skin or elsewhere |
| 9. | Infection with normally harmless tuberculosis-like bacteria |
| 10. | A family history of PID |

**Supplementary Table 6. New proposal of JMF warning signs for adults IEI.**

| **Warning signs in adults** | |
| --- | --- |
| 1. | ≥ 2 new ear infections within 1 year |
| 2. | ≥ 2 new sinus infections within 1 year, in the absence of allergy |
| 3. | 1 pneumonia per year for > 1 year |
| 4. | Chronic diarrhea with weight loss |
| 5. | Recurrent viral infections (colds, herpes, warts, condyloma) |
| 6. | Recurrent need for IV antibiotics to clear infections |
| 7. | Recurrent, deep abscesses of the skin or internal organs |
| 8. | Persistent thrush or fungal infection on skin or elsewhere |
| 9. | Infection with normally harmless tuberculosis-like bacteria |
| 10. | A family history of PID |
| **11.** | **Immune dysregulation event (lymphoproliferation AND/OR autoimmunity)** |

**Supplementary Table 7. Sensitivity, specificity, positive predictive value, and negative predictive value for JMF warning signs and updated JMF warning signs.**

|  | **Sensitivity (%)** | **Specificity (%)** | **PPV (%)** | **NPV (%)** |
| --- | --- | --- | --- | --- |
| JMF warning signs | 54.55 | 44.96 | 25.26 | 74.36 |
| JMF warning sign + Immune dysregulation | 93.18** | 24.81** | 29.71 | 91.43* |

*-*p* < 0.05; **-*p* < 0.001; Explanation of abbreviations—PPV, positive predictive value; NPV, negative predictive value

**Supplementary Table 8. Interpretation of variants according to ACMG classification criteria.**

| **Patient ID** | **Gene** | **Variant (cDNA)** | **Effect** | **ACMG classification** | **ACMG criteria** |
| --- | --- | --- | --- | --- | --- |
|  |  |  |  |  |  |
| IEI-1 | *ADA2* | c.1348G>A | p.Gly450Arg | Likely Pathogenic | PM1 (moderate) PM2 (moderate)  PP3 (supporting) PP4 (supporting) |
| IEI-2 | *BTK* | c.895-11C>A | Exon 11 deletion | Likely Pathogenic | PS3 (strong)  PM2 (moderate)  PP3 (supporting) PP4 (supporting) PP5 (supporting) |
| IEI-3 | *C8B* | c.1126C>T | p.Arg376Ter | Pathogenic | PVS1 (very strong) PM2 (moderate)  PP5 (supporting) |
|  |  | c.205C>T | p,Arg69Ter | Pathogenic | PVS1 (very strong) PM2 (moderate) PP5 (supporting) |
| IEI-4 | *CD8A* | c.272G>A | p.Gly91Asp | VUS | PM2 (moderate)  PP3 (supporting) PP4 (supporting) |
| IEI-5 | *CTLA4* | c.568-2A>G | ND | Likely Pathogenic | PVS1 (very strong) PM2 (moderate) |
| IEI-6 | *CTLA4* | c.425G>A | p.Gly142Asp | Likely Pathogenic | PS2 (strong)  PM1 (moderate) PM2 (moderate) |
| IEI-7 | *CTLA4* | c.253T>C | p.Cys85Arg | Likely Pathogenic | PM1 (moderate) PM2 (moderate)  PP3 (supporting)  PP4 (supporting) |
| IEI-8.1 | *ELANE* | c.133G>T | p.Val45Leu | Likely Pathogenic | PM1 (moderate) PM2 (moderate) PP3 (supporting)  PP4 (supporting) PP5 (supporting) |
| IEI-8.4 | *ELANE* | c.133G>T | p.Val45Leu | Likely Pathogenic | PM1 (moderate) PM2 (moderate)  PP3 (supporting) PP4 (supporting) PP5(supporting) |
| IEI-9.1 | *FAS* | c.632dupA | p.Ser212IlefsTer117 | Likely Pathogenic | PVS1 (very strong) PM2 (moderate) |
| IEI-9.2 | *FAS* | c.632dupA | p.Ser212IlefsTer117 | Likely Pathogenic | PVS1 (very strong) PM2 (moderate) |
| IEI-10 | *FAS* | c.753dupG | p.Asn252GlufsTer5 | Likely Pathogenic | PVS1 (very strong) PM2 (moderate) |
| IEI-11.1 | *FAS* | c.905_924dupAAAATTCAAACTTCAGAAAT | p.Glu309LysfsTer38 | Likely Pathogenic | PVS1 (very strong) PM2 (moderate) PP5 (supporting) |
| IEI-11.2 | *FAS* | c.905_924dupAAAATTCAAACTTCAGAAAT | p.Glu309LysfsTer38 | Likely Pathogenic | PVS1 (very strong) PM2 (moderate) PP5 (supporting) |
| IEI-12 | *FAS* | c.686T>A | p.Leu229Ter | Pathogenic | PVS1 (very strong) PM2 (moderate) PP5 (supporting) |
| IEI-13 | *FAS* | c.749G>A | p.Arg250Gln | Likely Pathogenic | PM1 (moderate) PM2 (moderate)  PM5 (moderate)  PP3 (supporting)  PP5 (supporting) |
| IEI-14 | *FAS* | c.679delG | p.Val227fsTer229 | Pathogenic | PVS1 (very strong) PM2 (moderate) PP5 (supporting) |
| IEI-15 | *FAS* | c.979T>G | p.Ile246Ser | Pathogenic | PS3 (strong)  PM1 (moderate) PM2 (moderate)  PM5 (moderate) PP3 (supporting) |
| IEI-16 | *FAS* | c.580delG | p.Glu194LysfsTer22 | Pathogenic | PVS1 (very strong) PM2 (moderate) PP5 (supporting) |
| IEI-17 | *FASL* | c.740C>A | p.Ala247Glu | Likely Pathogenic | PS3 (strong)  PM2 (moderate)  PP3 (supporting) PP4 (supporting) |
| IEI-18 | *GATA2* | c.913C>G | p.Leu305Val | Likely Pathogenic | PM1 (moderate) PM2 (moderate)  PP3 (supporting)  PP4 (supporting) |
| IEI-19 | *GATA2* | c.869C>A | p.Ser290Ter | Pathogenic | PVS1 (very strong) PM2 (moderate)  PP5 (supporting) |
| IEI-20 | *GATA2* | c.708delC | p.Met236IlefsTer325 | Pathogenic | PVS1 (very strong) PM2 (moderate)  PP5 (supporting) |
| IEI-21 | *GATA2* | c.1061C>T | p.Thr354Met | Likely Pathogenic | PM1 (moderate)  PM2 (moderate) PM5 (moderate) PP3 (supporting)  PP5 (supporting) |
| IEI-22 | *LIG4* | c.833G>A | p.Arg278His | Likely Pathogenic | PM1 (moderate)  PM2 (moderate) PM5 (moderate) PP3 (supporting)  PP5 (supporting) |
| IEI-23 | *MAGT1* | c.530delA | p.Asn177IlefsTer4 | Likely Pathogenic | PVS1 (very strong) PM2 (moderate) |
| IEI-24 | *NFKB1* | c.1597C>T | p.Gln533Ter | Likely Pathogenic | PVS1 (very strong) PM2 (moderate) |
| IEI-25 | *NFKB1* | c.1110_1119delTTTTTCGGAT | p.Asn370fsTer470 | Likely Pathogenic | PVS1 (very strong) PM2 (moderate) |
| IEI-26.1 | *PGM3* | c.1438_1442delTTAAG | p.Leu480SerfsTer10 | Pathogenic | PVS1 (very strong)  PM2 (moderate)  PP4 (supporting)  PP5 (supporting) |
|  |  | c.1475C>T | p.Thr492Ile | Likely Pathogenic | PM2 (moderate) PM3 (moderate)  PP3 (supporting)  PP4 (supporting) PP5 (supporting) |
| IEI-26.3 | *PGM3* | c.1438_1442delTTAAG | p.Leu480SerfsTer10 | Pathogenic | PVS1 (very strong)  PM2 (moderate)  PP4 (supporting)  PP5 (supporting) |
|  |  | c.1475C>T | p.Thr492Ile | Likely Pathogenic | PM2 (moderate) PM3 (moderate) PP3 (supporting)  PP4 (supporting) PP5 (supporting) |
| IEI-27 | *PIK3R1* | c.1425+1G>T | Exon 11 deletion | Pathogenic | PVS1 (very strong) PM2 (moderate)  PP5 (supporting) |
| IEI-28 | *PIK3R1* | c.1425+2delT | ND | Likely Pathogenic | PVS1 (very strong) PM2 (moderate)  PP5 (supporting) |
| IEI-29.1 | *RAB27A* | c.227C>T | p.Ala76Val | Likely Pathogenic | PM1 (moderate) PM2 (moderate)  PP3 (supporting) PP5 (supporting) |
| IEI-29.3 | *RAB27A* | c.227C>T | p.Ala76Val | Likely Pathogenic | PM1 (moderate) PM2 (moderate)  PP3 (supporting) PP5 (supporting) |
| IEI-30 | *STAT3 LOF* | c.1311C>A | p.His437Gln | Likely Pathogenic | PM2 (moderate)  PM5 (moderate) PP3 (supporting) PP5 (supporting) |
| IEI-31 | *STAT3 LOF* | c.1863C>G | p.Phe621Leu | Pathogenic | PS1 (strong) PM1 (moderate) PM2 (moderate)  PM5 (moderate) PP3 (supporting) |
| IEI-32 | *TAP1* | c.2059G>T | p.Glu687Ter | Likely Pathogenic | PVS1 (very strong) PM2 (moderate) |
| IEI-33 | *TBX1* | del22q11.2 | ND | Pathogenic | PVS1 (very strong) PM2 (moderate) PM6 (moderate) |
| IEI-34 | *TBX1* | del22q11.2 | ND | Pathogenic | PVS1 (very strong) PM2 (moderate) PM6 (moderate) |
| IEI-35.1 | *TET2* | c.1793delA | p.Asn598IlefsTer3 | Likely Pathogenic | PVS1 (very strong) PM2 (moderate) |
|  |  | c.277G>T | p.Gly93Ter | Likely Pathogenic | PVS1 (very strong) PM2 (moderate) |
| IEI-35.3 | *TET2* | c.1793delA | p.Asn598IlefsTer3 | Likely Pathogenic | PVS1 (very strong) PM2 (moderate) |
| IEI-36 | *TET2* | del724 Kb | ND | Pathogenic | PVS1 (very strong) PM6 (moderate) PP4 (supporting) |
| IEI-37 | *TLR7* | c.2050A>T | p.Lys684Ter | Pathogenic | PVS1 (very strong) PM2 (moderate)  PP4 (supporting) PP5 (supporting) |
| IEI-38 | *TNFRSF13B* | c.198C>A | p.Cys66Ter | Pathogenic | PVS1 (very strong) PM2 (moderate)  PP5 (supporting) |

**PM1**: Located in a mutational hot spot and/or critical and well-established functional domain without benign variation. **PM2**: Absent from controls (or at extremely low frequency if recessive) in Exome Sequencing Project, 1000 Genomes Project, or Exome Aggregation Consortium. **PM3**: For recessive disorders, detected in trans with a pathogenic variant. **PM5**: Novel missense change at an amino acid residue where a different missense change determined to be pathogenic has been seen before. **PM6**: Assumed de novo, but without confirmation of paternity and maternity. **PP3**: Multiple lines of computational evidence support a deleterious effect on the gene or gene product. **PP4**: Patient’s phenotype or family history is highly specific for a disease with a single genetic etiology. **PP5**: Reputable source recently reports variant as pathogenic, but the evidence is not available to the laboratory to perform an independent evaluation. **PS1:** Same amino acid change as a previously established pathogenic variant regardless of nucleotide change. **PS2**: De novo (both maternity and paternity confirmed) in a patient with the disease and no family history. **PS3:** Well-established in vitro or in vivo functional studies supportive of a damaging effect on the gene or gene product.**PVS1:** Null variant in a gene where LOF is a known mechanism of disease.

**PM1 (moderate) code was added to:**

**ADA2:** c.1348G>A: Hot-spot of length 17 amino-acids has 13 missense/in-frame variants (4 pathogenic variants, 9 uncertain variants and no benign).

**CTLA4:** c.425G>A: Hot-spot of length 17 amino-acids has 15 missense/in-frame variants (5 pathogenic variants, 10 uncertain variants and no benign).

**CTLA4:** c.253T>C: UniProt protein CTLA4_HUMAN domain 'Ig-like V-type' has 58 missense/in-frame variants (8 pathogenic variants, 50 uncertain variants and no benign). This position participates in a disulfide bond in a Ig-like domain.

**ELANE:** c.133G>T: pathogenic variants are in exon 2 and close to this variant (see Figure 1 in the reference 43 of the manuscript).

**FAS:** c.749G>A (Exon 9) and c.979T>G (Exon 9): Hot-spot of length 17 amino-acids has 18 missense/in-frame variants (8 pathogenic variants, 10 uncertain variants and no benign). UniProt protein TNR6_HUMAN domain 'Death' has 71 missense/in-frame variants (27 pathogenic variants, 44 uncertain variants and no benign). UniProt protein TNR6_HUMAN region of interest 'Interaction with CALM' has 25 missense/in-frame variants (9 pathogenic variants, 16 uncertain variants and no benign). UniProt protein TNR6_HUMAN region of interest 'Interaction with HIPK3' has 86 missense/in-frame variants (27 pathogenic variants, 57 uncertain variants and 2 benign variants).

**GATA2:** c.913C>G: UniProt protein GATA2_HUMAN zinc finger domain 'GATA-type 1' has 12 missense/in-frame variants (4 pathogenic variants, 8 uncertain variants and no benign).

**GATA2:** c.1061C>T: UniProt protein GATA2_HUMAN zinc finger domain 'GATA-type 2' has 50 missense/in-frame variants (34 pathogenic variants, 16 uncertain variants and no benign).

**LIG4:** c.833G>A: Hot-spot of length 17 amino-acids has 11 missense/in-frame variants (4 pathogenic variants, 7 uncertain variants and no benign). Position 278 is a binding site.

**RAB27A:** c.227C>T: Hot-spot of length 17 amino-acids has 6 missense/in-frame variants (4 pathogenic variants, 2 uncertain variants and no benign). Positions 74-78 belong to a binding site.

**STAT3:** c.1863C>G:UniProt protein STAT3_HUMAN domain 'SH2' has 79 missense/in-frame variants (42 pathogenic variants, 35 uncertain variants and 2 benign variants).

**PS3 (strong) code was added to:**

**BTK:** c.895-11C>A: A RT-PCR assay demonstrated that the variant c. 895-11C>A in BTK gene produced an exon skipping (reference 40 of the manuscript).

**FAS:** c.979T>G: Our group demonstrated by functional studies that T-cell blasts from the patient were completely resistant to the effects of the cytotoxic anti-Fas mAb CH-11, confirming a defective Fas-mediated apoptosis (reference 31 of the manuscript).

**FASL**: c.740C>A: Our group demonstrated by several functional studies an impaired FasL-induced cytotoxicity in the patient, confirming a defective activation-induced cell death (reference 19 of the manuscript).

**PP4 (supporting) code was added to:**

ADA2 (IEI-1): The phenotype of the patient is highly specific for ADA2 deficiency (ALPS-like phenotype, neurological affection, stomatitis, arthritis and vasculitis).

BTK (IEI-2): The phenotype of the patient is specific for BTK deficiency (Severe bacterial infections and absence of B lymphocytes).

CD8A (IEI-4): The phenotype of the patient is specific for CD8 deficiency with absence of CD8 lymphocytes.

CTLA4 (IEI-7): The phenotype of the patient is highly specific for CTLA-4 deficiency (Autoimmune cytopenias, enteropathy, interstitial lung disease, extra-lymphoid lymphocytic infiltration, recurrent infections).

ELANE (IEI-8): The phenotype of the patient is specific for ELANE deficiency (Severe congenital neutropenia).

FASL (IEI-17): The phenotype of the patient is highly specific for ALPS (Autoimmune cytopenias, splenomegaly and high counts of double negative T-cells).

GATA2 (IEI-18): The phenotype of the patient is highly specific for GATA2 deficiency (Susceptibility to mycobacteria and lymphedema) with absence of dendritic cells and NKbright subpopulation.

PGM3 (IEI-26): The phenotype of the patient is highly specific for hyper IgE syndrome (Severe atopy, immunodeficiency and high IgE levels).

TET2 (IEI-36): The phenotype of the patient is specific for TET2 deficiency (ALPS-like phenotype, immune dysregulation, high levels of vitamin B12 and IgG).


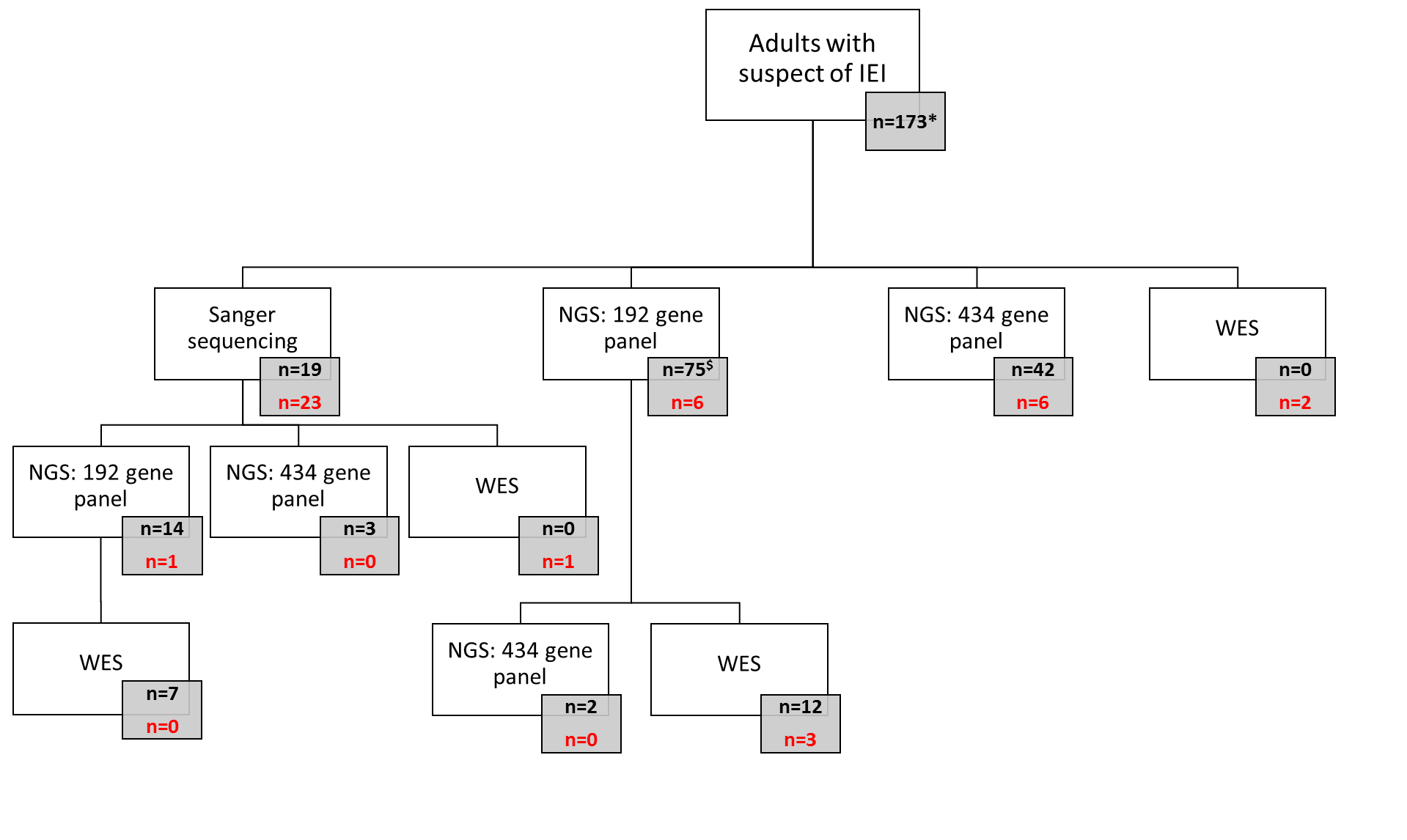


**Supplementary Figure 1:** Sequencing workflow follow by our center from 2005 to 2023. Patients without molecular diagnosis for IEI are highlighted in black. Patients with molecular diagnosis for IEI are highlighted in red.

*Patients IEI-34 was diagnosed by FISH.

^$^Patients IEI-36 was diagnosed by aCGH.


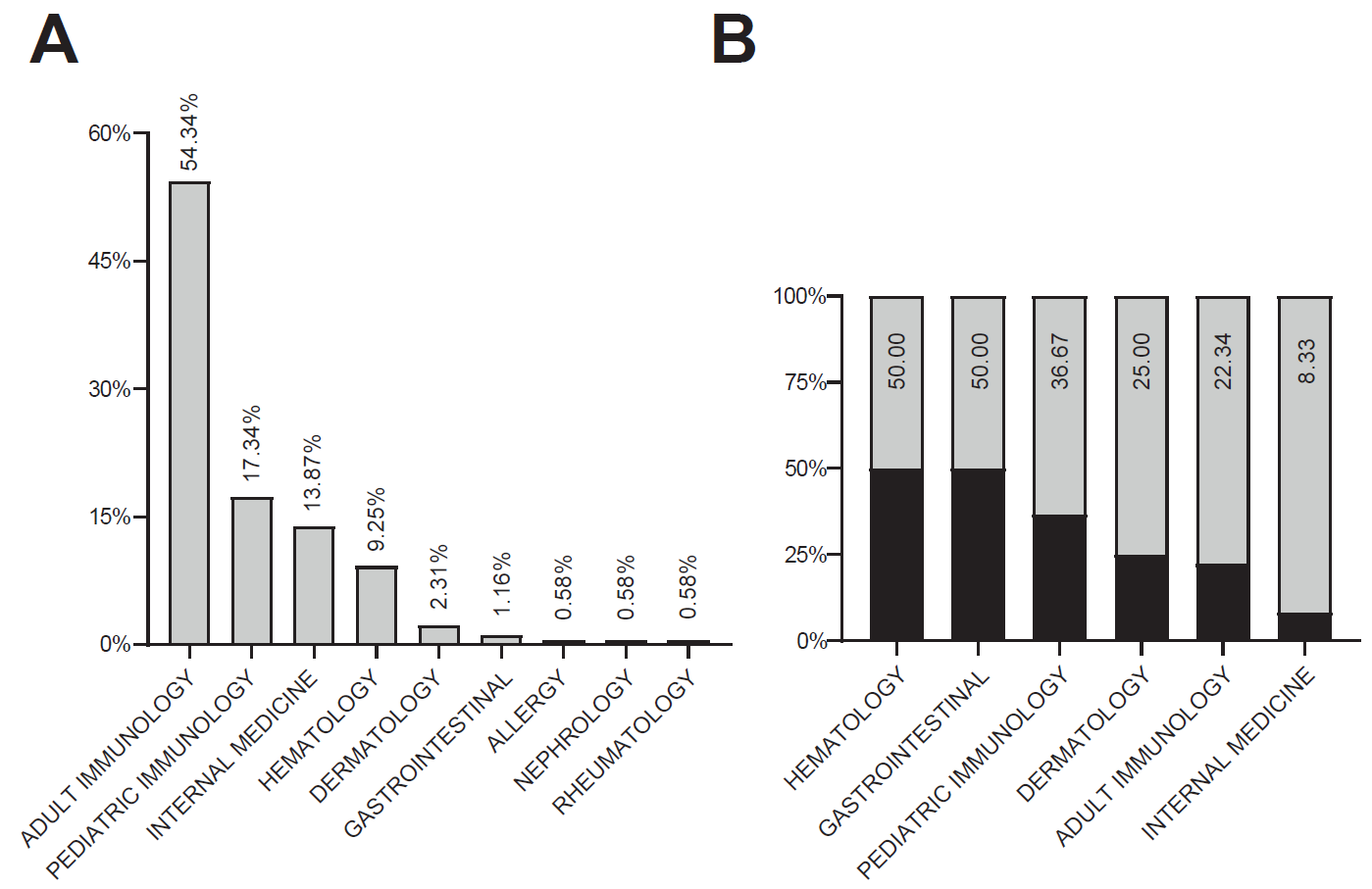


**Supplementary Figure 2:** **A.** Main requesting departments for molecular studies. **B.** Diagnostic rate as a function of the requesting department.

**Supplementary Figure 3:** Flow cytometry from IEI-4 that confirm the CD8 deficiency.
